# A Novel Nanopore-Based Genotyping System for Norovirus GII: Validation and Application to Pediatric Gastroenteritis Cases in Moscow, Russia

**DOI:** 10.3390/v17111440

**Published:** 2025-10-29

**Authors:** Ivan F. Stetsenko, Vasilina K. Lapshina, Polina A. Nikolaeva, Boris S. Gukov, Alexandr V. Chernitsov, Andrey R. Luparev, Ekaterina E. Davydova, Maria A. Gordukova, Elena V. Galeeva, Alina D. Matsvay, German A. Shipulin

**Affiliations:** 1 Federal State Budgetary Institution «Centre for Strategic Planning and Management of Biomedical Health Risks» of the Federal Medical Biological Agency, 119121 Moscow, Russiavlapshina@cspfmba.ru (V.K.L.); bgukov@cspfmba.ru (B.S.G.); edavydova@cspfmba.ru (E.E.D.);; 2 G. Speransky Children’s Hospital No. 9, 123317 Moscow, Russia; gordukovama@zdrav.mos.ru (M.A.G.);

**Keywords:** norovirus, nanopore sequencing, retrospective studies, Russia, gastroenteritis, computational biology, polymerase chain reaction

## Abstract

Norovirus is a leading cause of acute gastroenteritis, with genogroup II (NoV-GII) being predominant. This study presents a novel genotyping system for norovirus GII, combining long-range PCR to amplify the complete ~3.2 kb RdRp–VP1 region with Nanopore sequencing and a dedicated bioinformatics pipeline. This approach provides comprehensive, high-resolution genomic data, representing a significant advancement over conventional methods. Encompassing the recombination site and the full lengths of both genotyping regions in a single amplicon enables sensitive detection of mixed infections and recombinant variants, even at concentrations as low as 1% for minor genotypes. We validated the system through retrospective analysis of 115 pediatric acute gastroenteritis cases in Moscow, Russia (2021–2025) with a high viral load of NoV-GII, selected from 3061 screened stool samples. The analysis revealed the predominant circulation of GII.4[P16] norovirus strains, while GII.17[P17] emerged as the second most prevalent genotype after 2021. In contrast, previously common genotypes GII.4[P31] and GII.3[P12] sharply declined. Mixed infections were found in 4% of cases. With the data obtained, we are doubling the number of long (>3000 bp) NoV-GII genomic sequences from Russia available in public databases and providing unique surveillance data for the Moscow region covering the last five years. The results establish the system as a robust framework for high-resolution surveillance, supporting timely detection of emerging strains and informed public health response.

## 1. Introduction

Acute gastroenteritis (AGE) is one of the most common types of gastroenteritis worldwide and represents the second greatest burden among all infectious diseases, causing an estimated 145 million deaths annually as well as 895 million disability-adjusted life years [1]. AGE can be caused by a wide spectrum of pathogens, with enteric viruses accounting for up to 75% of infectious diarrhea cases [2]. Human noroviruses cause epidemic gastroenteritis in all age groups and have been associated with high-profile outbreaks in hospitals, nursing homes, cruise ships, etc. [3,4,5]. Each year, noroviruses are estimated to cause approximately 685 million cases of acute gastroenteritis, including 200 million cases among children under five years of age and 50,000 child deaths, mostly in developing countries [6].

Noroviruses are single-stranded RNA viruses of the *Caliciviridae* family, characterized by high genetic variability as well as the capability for recombination [7]. The *Norovirus* genus is classified into 10 genogroups (GI-GX), with GI, GII, GIV, GVIII and GIX being associated with human infections [8]. These genogroups are further divided into genotypes based on double systematics—RdRp based (at least 60 genotypes are registered) and capsid based (49 genotypes) [8,9]. Genogroup II (NoV-GII) is the most common and epidemiologically significant for humans [10], and genotype GII.4 is associated with more severe clinical manifestations of the disease [11,12]. Comprehensive genotyping of norovirus GII necessitates dual-segment analysis of the full-length VP1 capsid protein gene (ORF2; ~1600 bp) and the RNA-dependent RNA polymerase domain (ORF1; ~750 bp) within the RdRp region [8].

Recent epidemiological surveillance data indicate a rising incidence of norovirus infections in Russia, particularly among pediatric patients [13,14,15,16,17]. Noroviruses currently represent the second most prevalent viral etiology of acute gastroenteritis with confirmed pathogen identification [16]. National monitoring programs systematically characterize circulating norovirus genotypes and genogroups from outbreak clusters [18], tracking the viral epidemiology and emergence of novel variants.

Conventional norovirus GII surveillance systems [9,18,19,20,21,22] have been instrumental for routine genotyping but remain limited in their ability to resolve complex infections and recombinant forms. Consensus-based sequencing approaches tend to mask minority variants present in heterogeneous samples, which can obscure the true genomic diversity of circulating strains. These methodological gaps significantly compromise surveillance efforts during norovirus outbreaks, where accurate discrimination between true recombinants and mixed infections carries important epidemiological implications for tracking transmission pathways and viral evolution.

Previously published norovirus protocols [9,18,19,20,21,22] rely on amplification of short fragments (500–700 bp) that do not encompass the RdRp–VP1 recombination site, thus precluding direct identification of recombinant variants. While Sanger sequencing of separate polymerase and capsid fragments provides reliable genotyping [9,22], but cannot resolve mixed infections or detect minor variants due to consensus-based signal averaging. Although suitable for validation, these short fragments are suboptimal for comprehensive analysis. More advanced approaches, such as hybrid capture sequencing [23], enable full-genome recovery but are often too technically demanding, time-consuming, and costly for routine high-throughput surveillance.

This study aimed to develop a high-resolution norovirus genotyping system by combining long-range PCR amplification of the complete RdRp–VP1 region with Oxford Nanopore sequencing and a dedicated bioinformatics pipeline. This integrated approach enables precise genotyping and the sensitive detection of recombinant and mixed-genotype variants. In contrast to conventional methods, our technique simplifies workflow by generating the complete target in a single amplicon, thereby streamlining library preparation, reducing costs, and proving highly suitable for routine surveillance. The system was validated using clinical specimens from a pediatric acute gastroenteritis cohort in Moscow, Russia (2019–2025). Currently, only 91 long (>3000 bp) NoV-GII sequences from Russia are available in public databases; with the data obtained, we are doubling this number and providing unique surveillance data for the Moscow region covering the last five years, which is of separate importance, since no genomic NoV-GII data from Moscow is available in the public databases for this period.

## 2. Materials and Methods

### 2.1. Design of ONT Compatible NoV-GII Genotyping Primers

For NoV-GII specific genotyping primers design, genomic sequences of Norovirus GII (NCBI:txid122929) longer than 7000 nucleotides (indicating complete and nearly complete genomes) were downloaded from NCBI GenBank [24], resulting in 2895 sequences. A multiple sequence alignment was built using MAFFT [25]. Primer thermodynamic properties were screened using Primer3 [26]; coverage of the reference database of Norovirus GII genomic sequences was performed utilizing BLASTn v2.16.0 [27] for finding possible primer annealing sites. All primers were synthesized by Genterra (Moscow, Russia). Coverage screening was performed using the same 2895 sequence database that was used for primer design. Coverage of the reference database of Norovirus GII genomic sequences was performed utilizing BLASTn v2.16.0 [27] for finding possible primer annealing sites, using 8 as an initial word length and 15 as minimal alignment length counted from the 5′ end.

### 2.2. Pipeline for Automated Genotyping and Phylogenetics Analysis

Sequencing data were analyzed using the developed NorotyperII pipeline [28], which was implemented with Nextflow workflow description language (v25.04.7) [29].

Initial basecalling was performed using Dorado (v7.2.13) (Oxford Nanopore Technologies, Oxford, UK) in *super* mode. The pipeline processed base-called reads through the following stages, beginning with adapter trimming and quality control using Porechop v0.2.4 [30] to remove low-quality regions and adapters. Next, reads shorter than 3600 nucleotides were filtered out using trimmomatic (v0.39) [31]. Reads were clustered by similarity, and a consensus sequence was constructed for each resulting cluster of reads using Amplicon sorter v [32] with an expected maximum length of 3600 and similar_species_groups/similar_consensus of 96.0/auto and 98.0/98.0 for standard and sensitive modes, respectively.

Homologous sequences were identified using BLASTn (v2.17.0) [33] against reference databases for the RdRp and Capsid genes, as established by Chhabra et al. [8] and the NCBI non-redundant nucleotide database [24]. Hits were considered significant if the alignment length met the thresholds defined by Chhabra et al. [8]: ≥700 nucleotides for RdRp and ≥1400 nucleotides for Capsid. The BLASTn results for both genes were processed with a custom Python (v3.12) script and integrated across consensuses to determine composite genotypes. For each consensus, the best match based on total genotype coverage was selected. The corresponding RdRP and Capsid regions were extracted from the consensus sequences for phylogenetic analysis.

Phylogenetic analysis was conducted separately for each gene. Multiple sequence alignment was performed with MUSCLE (v5.3) [34], followed by alignment trimming with ClipKIT (v1.4.1) [35]. Maximum-likelihood phylogenetic trees were constructed using RAxML-NG (v1.2.2) [36] under the GTR + GI model of nucleotide substitution. The tree search utilized 30 parsimony-based and 30 random starting trees, with support assessed from 100 bootstrap replicates. Final tree visualization and annotation were performed using the iTOL web resource [37].

### 2.3. Sample Collection and Initial Screening

Clinical samples were collected from patients who were hospitalized from 2021 and 2025. In total, 3061 stool samples were obtained from pediatric patients, with one sample collected per patient. Written informed consent was obtained from each participant prior to sample collection.

Initial norovirus screening was conducted using the commercially available OKI-Screen-FL RT-PCR kit (AmpliSens, Moscow, Russia), following the manufacturer’s recommended protocol. This standardized assay enables sensitive detection of norovirus genomic material through fluorescence-based reverse transcription polymerase chain reaction (RT-PCR) technology.

Samples exhibiting high cycle threshold (Ct) values (>26), indicative of low viral load, were excluded from further genotyping analysis to ensure reliable amplification and sequencing results. As a result, 115 samples were successfully genotyped.

### 2.4. Target Enrichment and Nanopore Sequencing

Prior to target amplification, complementary DNA synthesis was performed using the Reverta kit (AmliTest, Moscow, Russia) and random primer according to the manufacturer’s instructions.

The complete ~3.2 kb RdRp–VP1 genomic region of norovirus GII was then amplified in a single PCR reaction using the primer pair noro_gt_fw (forward) and noro_gt_rv (reverse), specifically designed to flank this region and include the recombination site between the RdRp and VP1 genes. PCR amplification was performed with the BioMaster LR HS-PCR kit (Biolabmix, Novosibirsk, Russia) using the following reaction mixture: 10 µL of 2× PCR mix, 1 µL of each primer (10 µM), 2.5 µL of cDNA, and 5.5 µL of molecular-grade water. The reaction buffer contains a mixture of HS-Taq and Pfu polymerases, which enables efficient amplification of long DNA fragments.

Thermal cycling conditions were: 95 °C for 5 min; 45 cycles of 95 °C for 30 s, 55 °C for 30 s, and 70 °C for 4 min 30 s; followed by a final extension at 70 °C for 5 min.

Resulting amplicons were purified using KAPA Pure Beads (Roche Molecular Systems, Pleasanton, CA, USA) with a 0.7:1 bead-to-sample ratio and eluted in 10 µL. Barcoding PCR was performed using the BioMaster LR HS-PCR kit (Biolabmix, Russia) and PCR Barcoding Expansion 1–96 (ONT, Oxford, UK). The reaction mix contained 10 µL of 2× PCR mix, 1.25 µL of PCR barcode, and 7.5 µL of purified PCR product. Thermal cycling parameters were the same as for the primary amplification.

Post-barcoding cleanup was carried out with KAPA Pure Beads (Roche Molecular Systems, USA) at a 0.7:1 bead-to-sample ratio. Concentrations of barcoded PCR products were measured using the Qubit dsDNA HS Assay Kit (Thermo Fisher Scientific, Waltham, MA, USA). Libraries were pooled according to their concentrations to achieve equimolar representation in the final library. Adapter ligation with motor protein was performed using reagents from the NEBNext^®^ Companion Module for Oxford Nanopore Technologies^®^ Ligation Sequencing (NEB, Ipswich, MA, USA) together with the Ligation Sequencing DNA V14 Kit (ONT, UK), following the manufacturer’s instructions. Sequencing was conducted on a FLO-MIN114 flow cell (ONT, UK).

Alternatively, shorter overlapping fragments of the RdRp and VP1 regions can be obtained using primer pairs noro_gt_fw/noro_RdRp_rv and noro_VP1_fw/noro_gt_rv, respectively, under identical cycling conditions (annealing at 55 °C for 30 s). These shorter products are not required for routine nanopore sequencing but may be used for assay validation or Sanger sequencing if needed, either as a part of two separate reactions, amplifying VP1 and RdRp regions independently, or as internal primers during Sanger sequencing of the complete 3.2 kb RdRp–VP1 amplicon. Although these primer pairs were not employed for Sanger sequencing in the present study, they can be applied for this purpose when intermediate region verification is required. For these shorter amplicons, the use of long-range polymerase buffer is not essential.

## 3. Results

### 3.1. Development of ONT-Compatible NoV-GII Genotyping System

Our novel genotyping system enables complete amplification of the ~3.2 kb RdRp–VP1 genomic region in a single PCR reaction, spanning the critical recombination hotspot between these functional domains. To develop this system, degenerate primers were designed as described in Section 2.1 (“Design of ONT-Compatible NoV-GII Genotyping Primers”), based on multiple sequence alignments of publicly available Norovirus GII genomes.

Each primer sequence was extended at the 5′ end with an Oxford Nanopore-specific adapter, enabling direct, ligation-free PCR barcoding that significantly simplifies library preparation and reduces processing time compared to traditional ligation-based workflows.

**Figure 1:**
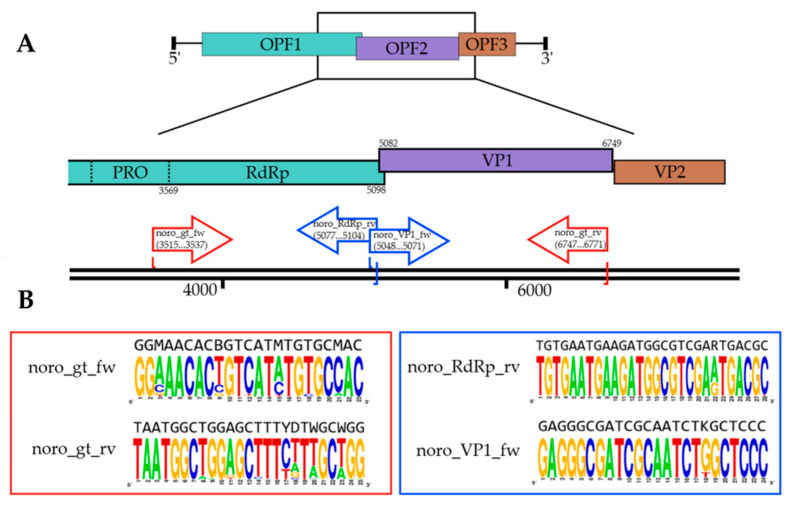
Primer design strategy for norovirus GII whole-genome genotyping. (**A**) Genomic organization of target regions showing primer positions relative to reference strain NC_044045. Red arrows indicate the primary primer pair spanning the complete ~3.2 kb RdRp-VP1 junction (positions 3569–6749), while blue arrows show nested PCR primers for independent amplification of RdRp and VP1 regions. (**B**) Sequence logos of primer binding sites demonstrate nucleotide variability at degenerate positions (indicated by standard IUPAC codes), with reverse primers displayed as their target strand sequences, illustrating the conservation profile of primer annealing sites across 487 GII reference sequences indicating position-specific variability.

Additionally, alternative primers were designed to target the RdRp and VP1 genes separately and can serve as internal primers for optional Sanger sequencing validation. The complete set of degenerate primers, including their nucleotide sequences, is presented in Table 1, and their genomic mapping is shown in Figure 1A, illustrating their positioning across the ~3.2 kb target region.

Table 2 summarizes the coverage of the Norovirus GII reference database, including the proportion of sequences with perfect matches, ≤1 mismatch, and no mismatches within the last five nucleotides at the 3′ end. Figure 1B demonstrates the conservation at primer binding sites in the form of a sequence logo, confirming the inclusivity of the design across diverse NoV-GII variants.

The developed primer system demonstrated high inclusivity across the currently available Norovirus GII genomic database (Table 2). By spanning the recombination hotspot between the RdRp and VP1 regions, the primers enable reliable detection of recombinant and mixed genotypes within a single amplicon. Moreover, the incorporation of ONT-compatible adapter tails allows direct PCR barcoding, significantly streamlining library preparation. Together, these features establish the primers as an efficient and versatile tool for high-throughput, full-length NoV-GII genotyping using nanopore sequencing.

### 3.2. Pipeline for Automated Genotyping and Phylogenetic Analysis

**Figure 2:**
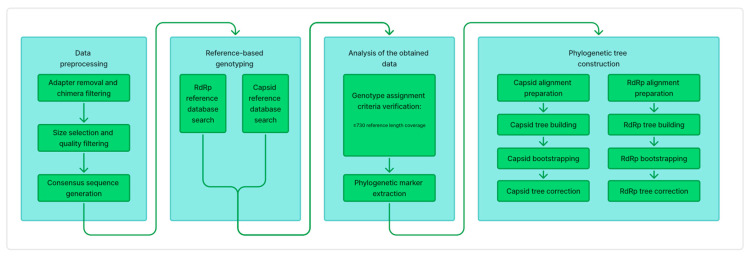
Schematic overview of the norovirus GII genotyping pipeline. The workflow processes Nanopore sequencing data through four key stages: (1) Data preprocessing. (2) Reference-based genotyping. (3) Analysis of the obtained data. (4) Phylogenetic tree construction.

The norovirus genotyping pipeline consists of four main stages (Figure 2). (1) Data preprocesing. This initial stage involves preprocessing and quality control of raw sequencing data. Reads are filtered, trimmed, and clustered to generate high-quality consensus sequences for downstream analysis. (2) Reference-based genotyping. In this stage, norovirus genotypes are determined through comparative analysis. Processed sequences are aligned against reference databases for two key genomic regions (capsid and RdRP) using local alignment algorithms to establish genotype classifications. We adapted the Preeti Chhabra classification system [8] to create a reference sequence database for genotype determination. The final database comprises 46 RdRP genotypes and 28 capsid genotypes. (3) Analysis of the obtained data. The genotyping results from both genomic regions are integrated and subjected to quality filtration based on alignment length and sequence identity. Following genotype confirmation, final sequence sets are prepared for phylogenetic analysis by extracting and aligning the target regions. (4) Phylogenetic tree construction. The final stage employs maximum likelihood methods to construct phylogenetic trees, enabling evolutionary analysis of norovirus strains. This phylogenetic reconstruction provides insights into genetic relationships between samples and their epidemiological connections.

We benchmarked the genotyping algorithm for two key parameters: genotype calling accuracy and sensitivity to minor genotype detection. The validation framework began with construction of a comprehensive reference dataset comprising all combinatorial variants of VP1 (capsid) and RdRp genotypes from known norovirus GII strains, including clinically relevant variants such as GII.4[P16], GII.3[P12], GII.2[P16], GII.4[P4], GII.4[P31], GII.6[P7], GII.7[P7], GII.10[P16], GII.16[P16], and GII.17[P17]. For sequencing simulation, we first processed reference templates by artificially fragmenting sequences at random positions and introducing Nanopore-characteristic errors using DeepSimulator [38], configured to match empirical error profiles (Q-score ≈ 18 equivalent). We then prepared 5000 synthetic read sets representing pairwise genotype mixtures across eight dilution gradients (1%, 2%, 4%, 8%, 16%, 24%, 32%, and 48% minor genotype frequency). This experimental design ensured balanced representation of all genotype combinations while systematically testing detection thresholds across clinically relevant concentration ranges.

**Figure 3:**
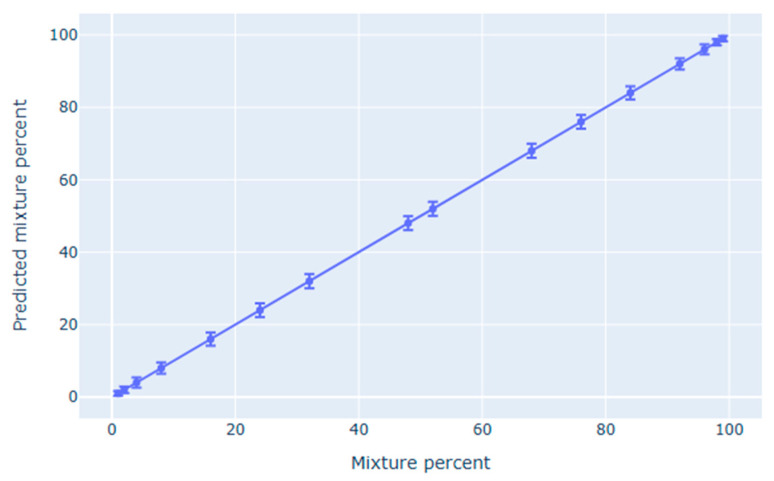
Accuracy of minor genotype quantification. The scatter plot demonstrates the strong linear correlation (R^2^ = 0.99) between experimentally simulated and computationally predicted proportions of minor genotypes in mixed norovirus samples. Confidence intervals represent the margin of error observed across all tested concentrations (1–48%) being less than ±2%.

The developed pipeline demonstrated exceptional performance in genotyping norovirus GII strains, achieving reliable detection of minor genotypes at concentrations as low as 1% (represented by more than 120 reads)—a significant improvement over conventional methods (Figure 3). Quantitative analysis revealed precise estimation of mixture ratios, with measurements consistently within ±2% of expected values and showing strong linear correlation (R^2^ > 0.99) across the entire tested range from 1–48% minor genotype concentration. The system successfully discriminated between all reference genotypes in the performance testing set, including clinically important variants such as epidemic GII.4 strains (P16), emerging GII.17[P17] types, and historically circulating GII.2[P16] variants. Performance metrics confirmed the method’s reliability, with 100% sensitivity (100% CI: 99.63–100%), 100% specificity (100% CI: 95.2–100%), and 100% positive predictive value (100% CI: 99.63–100%).

A critical challenge addressed by our pipeline involves accurately distinguishing samples containing either (1) identical capsid proteins with divergent polymerases or (2) matching polymerases with distinct capsid variants. This required particular attention given the inherent biological variation between these genomic regions—while capsid sequences exhibited substantial divergence (minimum: 0.13, median: 0.32, maximum: 0.37 nucleotide dissimilarity), polymerase sequences showed comparatively lower variation (range: 0.07–0.31).

To test this capability, we designed a comprehensive validation experiment. We systematically prepared artificial mixtures containing all possible combinations of: (i) identical capsid backbones paired with different polymerase genotypes, and (ii) conserved polymerase sequences coupled with divergent capsid variants. These test cases were subjected to realistic Nanopore sequencing simulations, including random read fragmentation and introduction of systematic errors (Q-score ≈ 18 equivalent).

**Figure 4:**
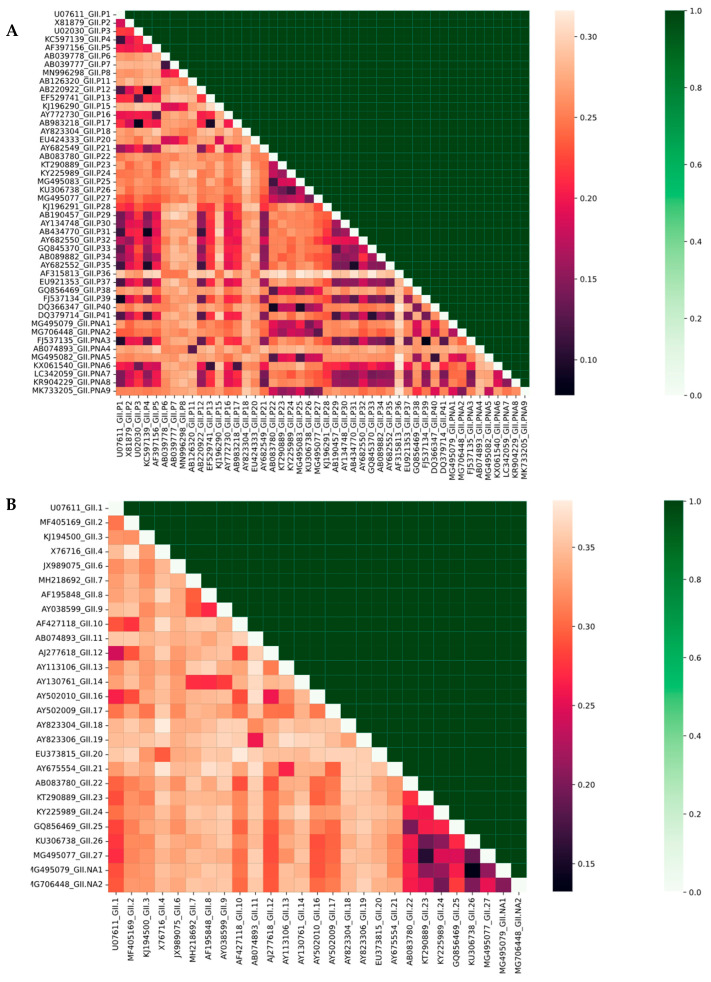
The pipeline’s genotyping accuracy against pairwise sequence divergence of different NoV-GII genotypes. The figure presents two symmetric heatmaps for (**A**) RdRP and (**B**) Capsid genotypes. The lower triangle of each matrix visualizes pairwise genetic distances (darker shades indicate higher sequence similarity). The upper triangle displays the results of in silico mixture experiments: for (**A**), two divergent RdRP sequences with a conserved capsid; for (**B**), two divergent capsid sequences with a conserved RdRP. Green cells indicate successful genotype discrimination by the pipeline, demonstrating high sensitivity, while light cells denote classification failure. The diagonal, representing zero divergence (self-comparison), provides a reference.

An in silico experiment was performed to calculate the ability of our pipeline to detect the presence of the secondary genotype in the artificially modeled sequencing data relative to the identity between the primary and secondary genotype sequences, since a lower degree of discrimination between highly similar sequences may lead to lower detection sensitivity of minor variants during in silico analysis. The analysis was performed separately for the RdRp and VP1 sequences, while keeping the second part of the region identical for both primary and secondary genotypes. As demonstrated in the accompanying Figure 4, our pipeline successfully resolved 100% of these challenging cases, correctly identifying both components even when two genotypes present in the mixture show a high degree of identity. This performance holds particular clinical relevance for detecting: (a) emerging recombinant strains, and (b) mixed infections involving closely related variants. The method’s robustness to sequencing artifacts was evidenced by consistent accuracy across the entire range of tested sequence dissimilarities.

These results confirm that the pipeline can accurately distinguish between recombinant and mixed genotypes, which is essential for reliable molecular surveillance and outbreak tracing.

### 3.3. In Vitro Studies: Genotyping Results, Phylogenetic and Epidemiological Analysis

A total of 3061 stool samples, collected from children presenting with acute gastroenteritis in Moscow, were screened for norovirus using real-time PCR. Among these, 443 (14.5%) samples tested positive. The annual distribution of positive cases was as follows: 26 in 2021 (18.7%), 96 in 2022 (12.6%), 75 in 2023 (11.6%), 194 in 2024 (16.2%), and 52 in 2025 (16.6%). To ensure robust genotyping results, samples exhibiting high Ct values (<26, suggestive of low viral load) in the Norovirus GII-specific RT-PCR were excluded from subsequent analysis. From the remaining positives, a total of 115 samples were successfully genotyped in full using the developed system.

**Figure 5:**
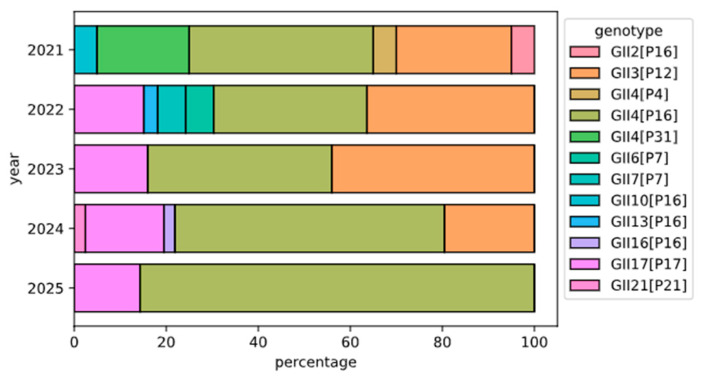
Temporal Distribution of Norovirus GII Genotypes. Stacked bar chart illustrating the annual prevalence of different Norovirus GII genotypes detected in pediatric patients with acute gastroenteritis in Moscow from 2021 to 2025 (*n* = 94). Each bar represents the proportional distribution of genotypes for a given year, summing to 100%. The specific genotype, defined by both capsid (e.g., GII.4) and polymerase (e.g., P16) regions, is indicated by the color key.

The genotypic analysis of selected samples delineated the distribution of circulating Norovirus GII genotypes among pediatric patients presenting with acute gastroenteritis between 2021 and 2025. The temporal progression and shifting prevalence of these genotypes are further illustrated in Figure 5.

The analysis revealed distinct patterns in the local circulation of norovirus genotypes. Consistent with global trends [39,40], the dominant genotype was GII.4[P16], which accounted for 63.8% of typed cases (*n* = 60). A notable shift in the molecular epidemiology was observed with the emergence of GII.17[P17] as the second most prevalent genotype (*n* = 17); this variant was absent in 2021 but subsequently detected in the following years. In contrast, the previously significant genotype GII.4[P31]—ranked second in prevalence in 2021—was not detected in subsequent years. Similarly, GII.3[P12], the third most common genotype in 2021, exhibited a marked decline in frequency over the study period and was absent from samples collected in 2025.

A total of five coinfection cases were identified over the study period: two in 2021, one in 2022, and two in 2024. One sample from 2021 contained a triple coinfection of the GII.4[P31], GII.4[P4], and GII.4[P16] genotypes. The remaining four cases involved dual coinfections, specifically: a combination of GII.3[P12] and GII.4[P16] in 2021; GII.13[P16] and GII.3[P12] in 2022; and two instances of GII.17[P17] and GII.4[P16] in 2024. For cases involving genotypes that differed in both their nucleocapsid and polymerase types, we analyzed individual sequencing reads to screen for evidence of viral recombination. However, no recombinant reads were detected in these samples, suggesting that ongoing recombination events did not occur in vivo in these particular patients. The lowest fraction of reads, classified as belonging to the minor genotype detected in the analyzed samples, was 3%, which is in line with the level of minor genotype detection estimated by in silico analysis.

**Figure 6:**
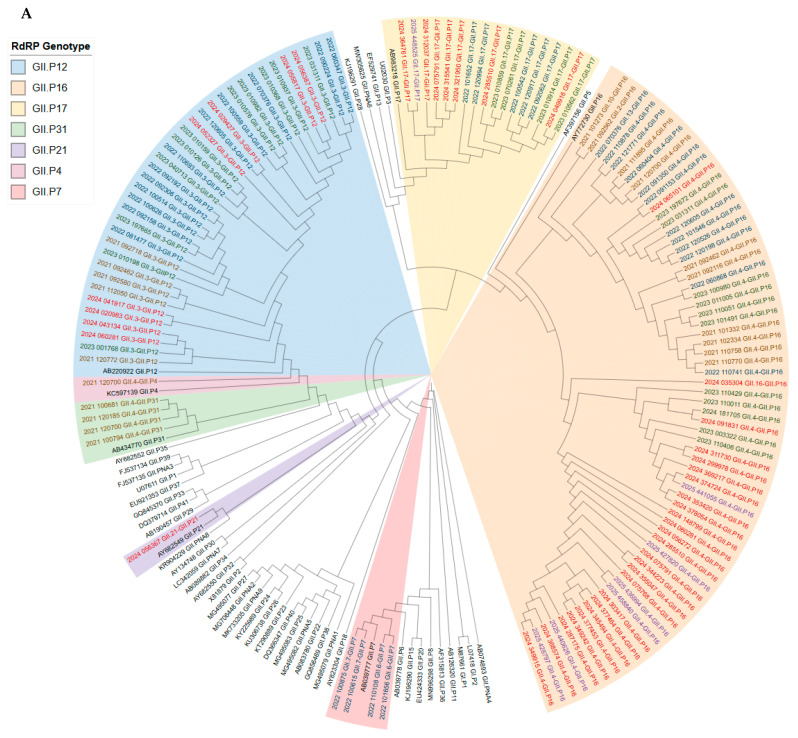

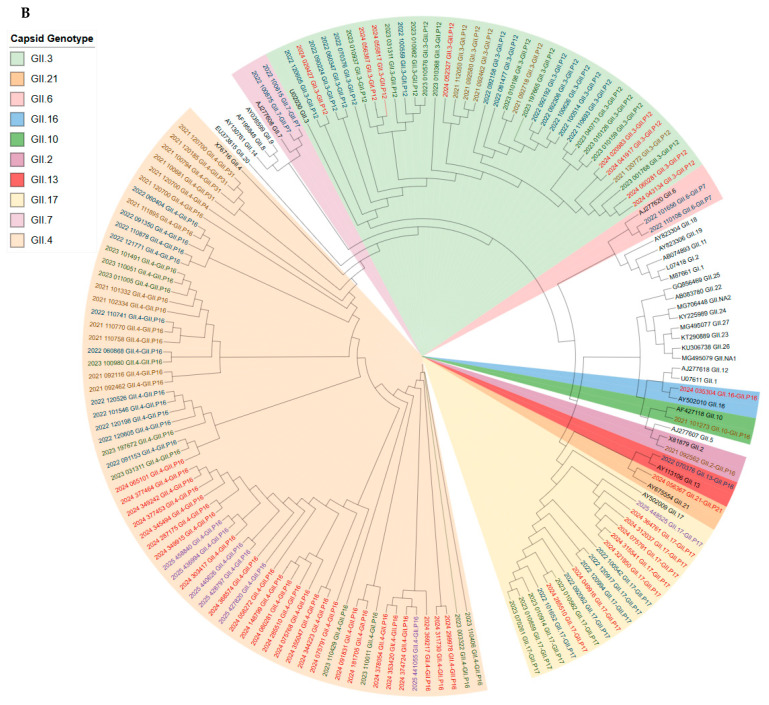
Phylogenetic analysis of norovirus sequences based on the RNA-dependent RNA polymerase (RdRp) (**A**) and capsid (**B**) regions. Major capsid genotypes are indicated by colored backgrounds and labels on the right, while the sampling year for each sequence is denoted by the font color. The consistent clustering of specific polymerase and capsid genotypes (e.g., GII.P16 with GII.4) supports strong genetic linkage, with noted exceptions indicating potential recombination events.

Phylogenetic analysis of both the RdRp and VP1 genes, shown on Figure 6, reveals that, within individual genotypes, sequences from earlier sampling years generally occupy more basal positions within the tree. This branching pattern indicates the gradual accumulation of genetic variation over time. A strong linkage was observed between specific polymerase and capsid genotypes across the study period: GII.P16 was predominantly associated with GII.4, GII.P17 with GII.17, and GII.P31 with GII.4. This consistent pairing suggests that recombination events shuffling the RdRp and VP1 genotypes are infrequent.

One notable exception to this trend was identified in sample 035304 (GII.P16-GII.16). In this case, the RdRp sequence clusters within a late-emerging clade observed exclusively from 2023 to 2025, while its VP1 sequence forms a distinct branch within the GII.16 genotype that is unique among the studied samples. This phylogenetic discordance is indicative of a relatively recent inter-genotypic recombination event.

## 4. Discussion

The developed norovirus GII genotyping system offers substantial improvements over conventional approaches. Traditional methods amplify only short (~560 bp) fragments spanning the recombination hotspot [41]. Such short-amplicon protocols often fail to capture complete recombinant structures and may misclassify mixed-genotype infections, particularly when one genotype predominates [18,19,20,21]. In addition, hybrid-capture and whole-genome sequencing approaches require high-quality RNA and are time-consuming and costly [23]. In contrast, our protocol achieves complete 3.2 kb coverage of the critical RdRp-VP1 region. This study evaluates a new methodological approach compared with traditional genotyping workflows, showing superior analytical resolution and quantitative accuracy in mixed-genotype samples. While current technologies can detect the presence of multiple genotypes in a sample, our system can confidently distinguish coinfections and recombinant variants, which is important in the context of global and regional trends in norovirus circulation [40]. This comprehensive sequencing enables simultaneous monitoring of both polymerase and capsid protein evolution with more accurate phylogenetic classification. The ORF1-ORF2 recombination hotspot represents a key evolutionary mechanism for GII noroviruses, with documented associations between specific recombination events and global variant emergence [42,43,44].

Compared to whole-genome sequencing approaches (~7 kb) that demand both high-quality intact RNA and genotype-specific primer sets [20], our method offers tolerance for partially degraded samples, comprehensive coverage using a single universal primer pair for most genogroup II representatives and flexible compatibility with both Oxford Nanopore (for rapid, high-throughput analysis) and Sanger sequencing (for cost-effective, targeted verification). This flexibility significantly enhances the method’s utility for routine surveillance and clinical diagnostics.

Application of our method to clinical specimens from pediatric acute gastroenteritis cases in Moscow demonstrated the predominant circulation of GII.4[P16] norovirus strains. These findings align with Russian national surveillance data showing P16 prevalence rates of 80% (2017) and 60% (2020) [17] as well as with global epidemiological trends where GII.4[P16] and its recombinant variants maintain dominance across multiple geographic regions [45,46,47]. Recent global surveillance studies demonstrate that the P16 polymerase has emerged as one of the most successful norovirus variants in circulation, effectively displacing previously dominant strains [9,48].

Recent epidemiological surveillance has revealed an emerging trend of GII.4[P16] decline coinciding with increased circulation of GII.3[P12] variants, which is the second most observed genotype in this study. Genomic characterization indicates that GII.3[P12] originated through intragenic recombination between GII.4[P12] and GII.3[P21] lineages [39], This recombinant variant exhibits an accelerated substitution rate in the RdRp gene, and critical amino acid substitutions at positions 385 and 406 of the VP1 capsid protein—key antigenic determinants that may influence host immune recognition [39].

The high prevalence of the GII.17[P17] genotype observed in this pediatric cohort presents a notable finding. Initially described as emerging in East Asia around 2014 [49], this variant was historically reported to exhibit higher prevalence in adult populations than in children, as documented in studies from Japan [50] and China [51]. The significant frequency of GII.17[P17] infections among children in Moscow may therefore represent an evolving epidemiological shift. This observation aligns with the recent global resurgence of GII.17[P17], which has been rapidly rising in incidence across the Americas and Europe since 2023 and is increasingly identified as a major cause of outbreaks [52]. Thus, while unusual in a historical context, the emergence of GII.17[P17] as the third most common genotype in this study reflects its changing age-specific epidemiology and indicates that Moscow is following this broader international trend.

Our data demonstrate consistent circulation of the P12 genotype across all study years except 2025 (possibly due to the small number of 2025 samples). Wave-like epidemiological pattern of GII.3[P12] is noted in national surveillance reports from Russia [17], which document periodic surges in P12 prevalence, particularly among pediatric populations. The recurrent emergence of this genotype suggests either cyclical population immunity patterns or ongoing adaptive evolution enabling intermittent selective advantages. Global epidemiological studies consistently identify GII.3[P12] noroviruses as important pediatric pathogens, capable of causing both sporadic pediatric cases and localized outbreaks [48]. These variants demonstrate widespread circulation across Asia and Europe [39,40], with their successful dissemination potentially attributable to enhanced transmissibility in young child populations, and/or improved immune evasion capabilities mediated by antigenic variation [44,53].

Apart from these three predominant genotypes, we have observed a number of rarer variants, including an apparent newly recombined GII.16[P16]. These finding highlights the importance of epidemiological monitoring, since another P16-bearing genotype, GII.2[P16], has previously caused an outbreak in Nizhny Novgorod in 2017 [54].

The epidemiological landscape in Nizhny Novgorod during 2020–2021 revealed an additional distinctive pattern, with GII.2[P16] accounting for a substantial proportion (34.3%) of circulating strains. This contrasts sharply with our Moscow dataset, where this genotype was detected in only a single case (2021). These findings underscore the necessity for decentralized surveillance systems to detect regionally emerging strains and guide localized public health responses.

This study has several important limitations that should be considered when interpreting the results. First, our exclusive focus on hospitalized cases may have introduced selection bias toward more clinically severe norovirus strains, potentially underestimating the diversity of circulating variants in the community. Second, the relatively small sample size limited our ability to detect rare genotypes and conclusively rule out random fluctuations in genotype prevalence. Since the developed system is directed towards the genotyping of G.II Norovirus in the samples for which its presence was already determined by another technique (e.g., RT-qPCR) rather than detecting it, diagnostic sensitivity and specificity of the system were out of scope of the study and were not analyzed. During this study, only samples with high viral load were chosen for the analysis. Third, the relatively large amplicon size (~3.2 kb) requires high-quality RNA templates, which may limit assay performance when working with highly degraded clinical material. These constraints highlight the need for future population-based studies with larger, more representative samples to validate our findings. Future research should prioritize comprehensive sampling encompassing outpatient cases and asymptomatic carriers to obtain a more representative profile of community norovirus circulation and advanced phylogenetic characterization of rare genotypes (e.g., GII.10[P16]) to elucidate their evolutionary origins and potential epidemiological significance.

Although GII.4[P16] remains globally predominant, emerging variants such GII.3[P12] and GII.17[P17] continue to play an important epidemiological role. The rapid evolutionary dynamics of noroviruses, driven by both recombination events and antigenic drift, necessitate sustained molecular epidemiological surveillance at national and global scales. Such studies would provide critical insights into norovirus transmission dynamics across the full clinical spectrum and identify emerging variants of concern.

Our results demonstrate that the developed genotyping system is an effective tool for norovirus molecular epidemiology surveillance, capable of rapidly detecting changes in circulating strains. Its enhanced resolution improves the identification of emerging variants and recombinants, thereby supporting timely public health interventions and informing outbreak prevention strategies. The system’s high resolution makes it particularly valuable for tracking viral evolution and strengthening genomic surveillance efforts. The sequencing data obtained during this study doubles the amount of available long genomic sequences of NoV-GII from Russia, and its simplicity and streamlined approach pave the way for more research in the area.

## Figures and Tables

**Table 1:** Sequences of primers designed for this study. Underlined region represents ONT adapter sequences used for ligation-free PCR barcoding.

Primer	Sequence
noro_gt_fw	TTTCTGTTGGTGCTGATATTGC- GGMAACACBGTCATMTGTGCMAC
noro_gt_rv	ACTTGCCTGTCGCTCTATCTTC- CCWGCWAHRAAAGCTCCAGCCATTA
noro_VP1_fw	TTTCTGTTGGTGCTGATATTGC- GAGGGCGATCGCAATCTKGCTCCC
noro_RdRp_rv	ACTTGCCTGTCGCTCTATCTTC- GCGTCAYTCGACGCCATCTTCATTCACA

**Table 2:** Fractions of sequences covered with no mismatches, no mismatches within terminal 5 3′ bases and with one mismatch.

Primer	Fraction of Sequences Covered with No Mismatches	Fraction of Sequences Covered with No Mismatches in Terminal 5 3′ Bases	Fraction of Sequences Covered with No More than 1 Mismatch
noro_gt_fw	72.5%	97.1%	92.1%
noro_gt_rv	68.0%	99.4%	91.2%
noro_VP1_fw	98.1%	98.3%	99.5%
noro_RdRp_rv	93.2%	98.7%	99.1%

## Data Availability

The original data presented in the study are openly available in NCBI GenBank database; accession number PX458462-PX458563, PX464553-PX464579. The developed genotyping software is freely available for non-commercial use in the GitHub repository at the following link: https://github.com/Laboratory-of-molecular-epidemiology/NorotyperII, accessed on 30 September 2025.
